# A new *Ceratomyxa* (Cnidaria: Myxosporea) infecting the ornamental fish species *Pterophyllum scalare* from the Amazon Region, Brazil

**DOI:** 10.1590/S1984-29612024075

**Published:** 2024-12-13

**Authors:** Rafaela Franco de Araújo, Abthyllane Amaral de Carvalho, Roger Leomar da Silva Ferreira, Saturo Cardoso Morais, Luize Cristine Pantoja dos Reis, Pedro Lucas dos Santos de Oliveira, Marcela Nunes Videira, Aldi Feiden

**Affiliations:** 1 Universidade Estadual do Oeste do Paraná – UNIOESTE, Toledo, PR, Brasil; 2 Universidade do Estado do Amapá – UEAP, Macapá, AP, Brasil; 3 Universidade Federal do Pará – UFPA, Belém, PA, Brasil; 4 Universidade Federal do Amapá – UNIFAP, Macapá, AM, Brasil

**Keywords:** Amapá, Cichlidae, aquariophilia, Myxozoa, parasite, phylogeny, Amapá, Cichlidae, aquariofilia, Myxozoa, parasito, filogenia

## Abstract

A new parasite of the Class Myxozoa is described in the gallbladder of the ornamental angelfish *Pterophyllum scalare*, in two municipalities in the state of Amapá, Brazil, based on morphological, morphometric and phylogenetic descriptions. From October 2022 to August 2024 fifty-five angelfish specimens were sampled in Macapá (n=10) and Tartarugalzinho (n=45). Slightly arched mixospores were observed by light microscopy and had characteristics consistent with those of the genus *Ceratomyxa*. These obtained an average length of 1.6 ± 0.2 µm and 11.5 ± 1.1 µm in thickness. The polar capsules were subspherical and 0.7 ± 0.1 µm long and 0.6 ± 0.1 µm wide, with 3 to 4 turns of the polar filament. Phylogenetic analysis showed that the new species is grouped in the family Ceratomyxidae, in addition to being positioned in the same subclade of freshwater ceratomyxids from the Brazilian Amazon, demonstrating that this species shares a common ancestor with its close relatives, based on geographic affinity. *Ceratomyxa tavariensis* n. sp. is the first species of the class Myxozoa described infecting angelfish in Brazil, and the thirteenth species of *Ceratomyxa* described in the country.

## Resumo

Um novo parasito da Classe Myxozoa é descrito na vesícula biliar do peixe de importância ornamental, angelfish *Pterophyllum scalare*, em dois municípios do estado do Amapá, Brasil, com base nas descrições morfológicas, morfométricas e filogenéticas. De outubro de 2022 até agosto de 2024, 55 espécimes de angelfish foram amostrados nos municípios de Macapá (n=10) e em Tartarugalzinho (n=45). Foi observado por microscopia de luz mixósporos levemente arqueados, com características consistentes com as do gênero *Ceratomyxa*, que obtiveram comprimento médio de 1,6 ± 0,2 µm e 11,5 ± 1,1 µm de espessura. As cápsulas polares foram subesféricas, tendo 0,7 ± 0,1 µm de comprimento e 0,6 ± 0,1 µm de largura, com 3 a 4 voltas do filamento polar. A análise filogenética mostrou que a nova espécie está agrupada na família Ceratomyxidae, além de estar posicionada no mesmo subclado de ceratomixídeos de água doce da Amazônia brasileira. Demonstra-se, assim, que esta espécie compartilha de um ancestral comum com seus parentes próximos, com base na afinidade geográfica. *Ceratomyxa tavariensis* n. sp. é a primeira espécie da classe Myxozoa descrita infectando angelfish no Brasil, e a décima terceira espécie de *Ceratomyxa* descrita no país.

## Introduction

The global trade in ornamental fish is worth around US$ 15–30 billion and involves more than 2,500 species, mainly freshwater tropical fish (Correa et al., 2024). In Brazil, this industry is supported by extractive fishing with wild fish stocks, with production in the Amazon basin being particularly important due to its high biodiversity of ichthyofauna (Araújo et al., 2020; Mattos et al., 2024; Ribeiro et al., 2024).

Ornamental fishing in the Amazon began to show signs of decline in the 2000s, due to the impacts of degradation of the freshwater environment, overfishing by fishermen, and biopiracy (Biondo & Burki, 2020; Sousa et al., 2021; Oliveira et al., 2023). This scenario poses threats to the sustainability of this activity, leading to the need to strengthen regulatory institutions to implement effective legislation (Sousa et al., 2021). In addition, there is a need to develop technologies for the conservation of natural stocks, drawing attention to the importance of knowledge about the health of ornamental fish in the region (Ladislau et al., 2020, Ribeiro et al., 2023; Mattos et al., 2021; Barros et al., 2023).

The angelfish or acará bandeira *Pterophyllum scalare* (Schultze, 1823) is a small species of the Cichlidae family, which is among the thirty freshwater species that dominate the world ornamental aquaculture market, gained popularity due to its attractive body pattern, peculiar coloration and special behavior (Dey, 2016; Jayalekshmi et al., 2017; Patil et al., 2015). The distribution of natural populations of this species covers countries in South America, such as Brazil, Colombia, French Guiana, Guyana, Peru and Suriname, with the angelfish originating from the Amazon basin (Froese & Pauly, 2024).

The class Myxozoa Grassé, 1970 (Kyger et al., 2021) constitutes a diverse clade of cnidarian endoparasites with complex life cycles, which commonly require annelids or bryozoans as invertebrate hosts and fish from marine, freshwater and terrestrial environments as vertebrate hosts (Okamura et al., 2018). Currently, approximately 2,600 species of myxozoans belonging to 67 genera have been described and known worldwide (Okamura et al., 2018).

*Ceratomyxa* Thélohan, 1892 is notable as the second largest genus of myxozoans (approximately 270 species described worldwide). They are Coelozoic parasites mainly of the gallbladder of marine fish (Eiras et al., 2018; Li et al., 2023), and approximately 10 freshwater species reported in hosts mainly from South America are derived from marine ancestors (Zatti et al., 2023). This genus is distinguished from other members of Myxosporea by being elongated, commonly in crescent or arched shapes. The length of the shell valve exceeds the axial diameter of the spore and each myxospore has two subspherical polar capsules of equal size (Lom & Dyková, 2006).

To date, the richness of myxozoans infecting freshwater ornamental fishes remains poorly explored, with scarce investigations of myxozoans infecting ornamental species in South America (Bittencourt et al., 2022; Mathews et al., 2016, 2017, 2018, 2020, 2022). This study describes a new species of *Ceratomyxa* infecting the gallbladder of *P. scalare* from the municipalities of Tartarugalzinho and Macapá, Amapá, in the Brazilian Amazon, an important region that has a diversity of highly valued commercial ornamental fish species for international markets.

## Material and Methods

### Sample collection and morphological and morphometric analyses

**Figure 1:**
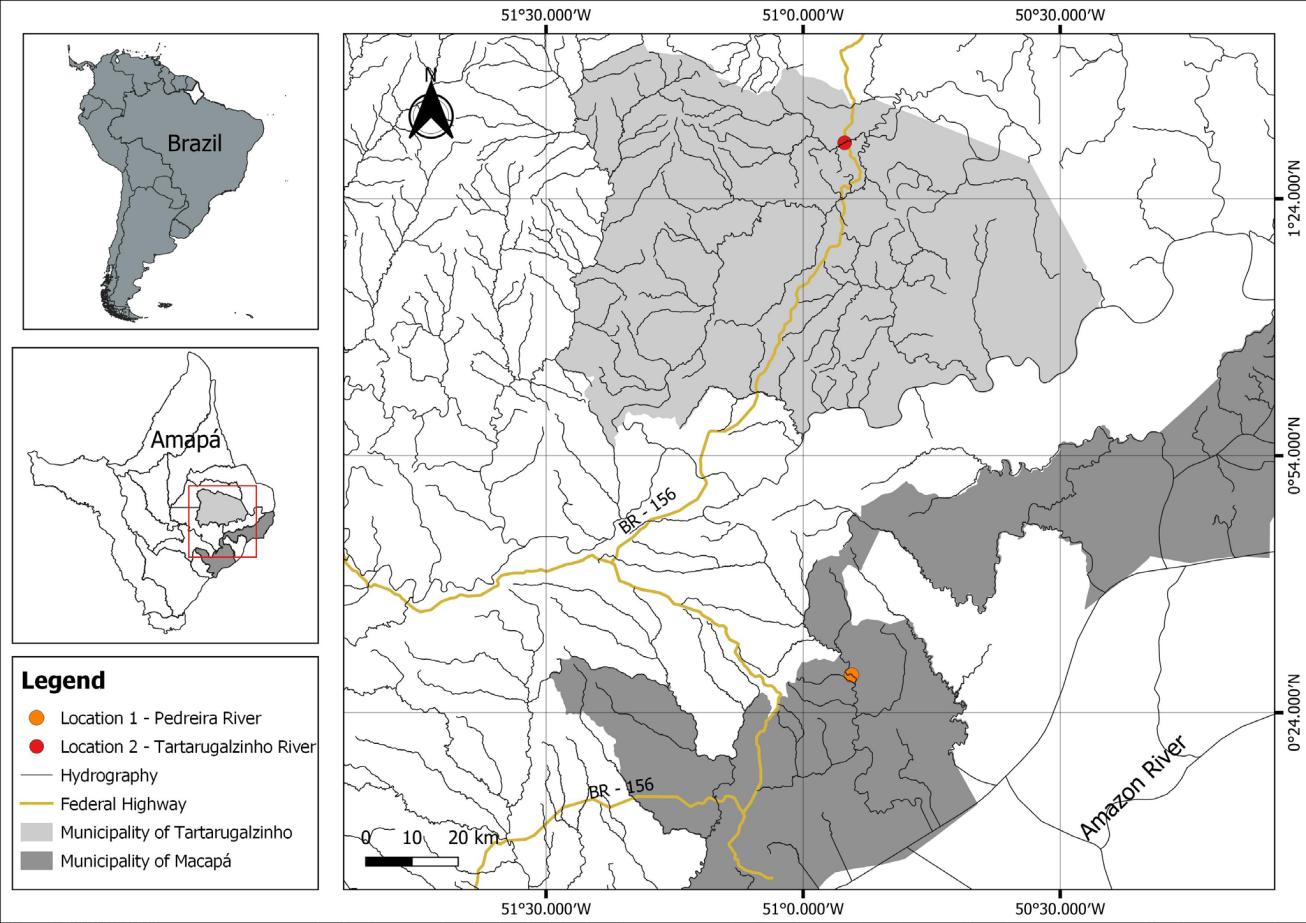
Location map of *Pterophyllum scalare* collections in the state of Amapá: Pedreira River (orange dot), municipality of Macapá and Tartarugalzinho River (red dot), municipality of Tartarugalzinho.

Samples were collected from 10 specimens of *P. scalare* (7.4 ± 1.7 g and 7.7 ± 0.7 cm) raised in 1m^3^ PVC (polyvinyl chloride) tanks with constant water renewal, from a laboratory located in Macapá-AP. These came from the Pedreira River (0°28'22.8”N 50°54'21.6”W), located in the Mangabeira community, a rural area of Macapá-AP. Additionally, 45 specimens of *P. scalare* (12.5 ± 5.6 g and 8.2 ± 0.9 cm) we obtainedfrom the Tartarugalzinho River (01°30'32.2”N 050°55'09.9”W), located in the municipality of Tartarugalzinho-AP, in the Brazilian Amazon (Figure 1). The Tartarugalzinho River is part of the Macari-Tartarugal Grande basin, which is of great importance for local fishing, and flows into the Atlantic Ocean.

The collections were carried out from October 2022 to August 2024, adopting the same methodological treatment from the capture process, transportation to analysis. To capture the fish, lines and hooks, cast nets and gillnets measuring 20 mm between knots were used to obtain a significant sample size. The animals were then transported to the Laboratory of Morphophysiology and Animal Health (LABMORSA) of the State University of Amapá (UEAP).

Freshly captured fish were anesthetized with tricaine methanesulfonate MS-222 (50 mg/L ^-1^), euthanized by neural myelotomy and necropsied. The fish were then measured (cm) and weighed (g). After biometry, the gallbladders were extracted, ruptured and the bile samples were analyzed under light microscopy to verify the presence of parasites. Plasmodia and parasite spores were photographed with a digital camera (Moticam 2300 3.0 M) attached to the microscope. The morphometry of the myxospores (µm) was obtained according to Matos et al. (2001) and analyzed according to Lom & Arthur (1989) and included spore length (SP), spore thickness (ST), polar capsule length (PCL), polar capsule width (PCW) expressed in μm, and posterior angle (PA) in degrees (°). The dimensions were made with 30 spores and included mean ± standard deviation. The prevalence (%) of infection was calculated according to Bush et al. (1997).

### Histological analyses

For the histological procedure, fragments of the gallbladder were collected and fixed in Davidson (95% alcohol, formaldehyde, acetic acid and water), dehydrated, and passed through increasing concentrations of alcohols (70%, 80%, 90%, absolute I, II and III), then diaphanized using xylene, infiltrated and embedded in paraffin blocks. Afterwards, they were sectioned using a microtomy technique to obtain 5 µm thick sections, stained with the Ziehl-Neelsen technique (Luna, 1968), analyzed and photographed under an optical microscope, to verify the presence of histopathological alterations.

### DNA extraction and amplification

Infected gallbladders were fixed in 80% ethanol for DNA extraction. DNA was extracted from these samples using the ReliaPrep gDNA Tissue Miniprep System (Promega, USA) following the manufacturer's protocol. Molecular analyses were based on 18S rDNA sequences amplified by nested PCR in a MyGene MG96G thermocycler (LongGene, China). The first round of amplification was performed using primers 18E (5'-CTGGTTGATCCTGCCAGT-3') (Hillis & Dixon, 1991) and 18R (5'-CTACGGAAACCTTGTTACG-3') (Whipps et al., 2003) with initial denaturation at 95 °C for 15 min, followed by 35 cycles of 95 °C for 1 min, 48 °C for 1.5 min and 72 °C for 2 min plus final extension at 72 °C for 10 min. A second round of amplification was then performed using the primer pairs 18E - MC3 (5'-GATTAGCCTGACAGATCACTCCACGA-3') (Molnár et al., 2002) and MC5 (5'-CCTGAGAAACGGCTACCACATCCA-3') (Molnár et al., 2002) - 18R with initial denaturation at 95 °C for 15 min, followed by 35 cycles of 95 °C for 30 s, 56 °C for 30 s, and 72 °C for 1 min plus final extension at 72 °C for 10 min. The polymerase chain reaction (PCR) was performed in a total volume of 25 µL containing 2.5 µL of buffer, 1.5 mM MgCl2, 0.2 mM dNTP (Sinapse Inc., Brazil), 0.3 µM of each primer, 1U of HOT FIREPol taq DNA Polymerase (Solis BioDyne, Estonia), 3.0/2.0 µL of template DNA and water for PCR until completing the final volume. The PCR products were visualized in 1.5% agarose gel in Tris-borate-EDTA buffer, stained with UniSafe Dye (UniScience, Brazil). Positive samples were sent for sequencing to ACTGene (Alvorada, RS, Brazil).

The *Ceratomyxa* sequences obtained were assembled and edited in Geneious® 7.1.3 software. BLASTn searches (Altschul et al., 1997) were performed on the NCBI nucleotide database in order to determine sequence similarity.

### Phylogenetic analyses

A database comprising 46 SSU-rDNA sequences of myxozoan fish parasite species was constructed according to the BLASTn search. *Ellipsomyxa tucujuensis* Ferreira, Silva, Carvalho, Bittencourt, Hamoy, Matos & Videira, 2021 was used as an outgroup. This database was aligned using the MUSCLE algorithm with its default parameters, in the Geneious 7.1.3 software (Kearse et al., 2012).

Bayesian inference (BI) analysis was conducted in MrBayes 3.2.7a (Ronquist & Huelsenbeck, 2003) through the CIPRES platform, with the evolution model (GTR + I + G) selected by jModelTest analysis, based on the lowest Bayesian information criterion (BIC) score. Posterior probabilities were based on 10 million generations via Markov Chain Monte Carlo (MCMC) algorithms. A consensus tree (majority rules) was estimated using the topologies (Miller et al., 2010). Genetic distance was analyzed through p-distance with the aid of the MEGA11 program, in which it was possible to establish the relationships of *Ceratomyxa* species. The phylogenetic tree generated was visualized in FigTree 1.3.1 software (Rambaut, 2020) and edited in CorelDraw 2019.

## Results

The occurrence of infection by mature myxospores was recorded in the gallbladder of 6 of 45 (13.3%) specimens of *P. scalare* collected in the Tartarugalzinho River and 3 of 10 specimens (30.0%) collected in the municipality of Macapá. The morphological characteristics of the collected myxospores were consistent with those of the genus *Ceratomyxa*.

### Taxonomic summary

Phylum Cnidaria Hatschek, 1888

Class Myxozoa Grassé, 1970 (Kyger et al., 2021)

Subclass Myxosporea Bütschli, 1881

Order Bivalvulida Shulman, 195

Family Ceratomyxidae Doflein, 1899

Genus *Ceratomyxa* Thélohan, 1892

**Figure 2:**
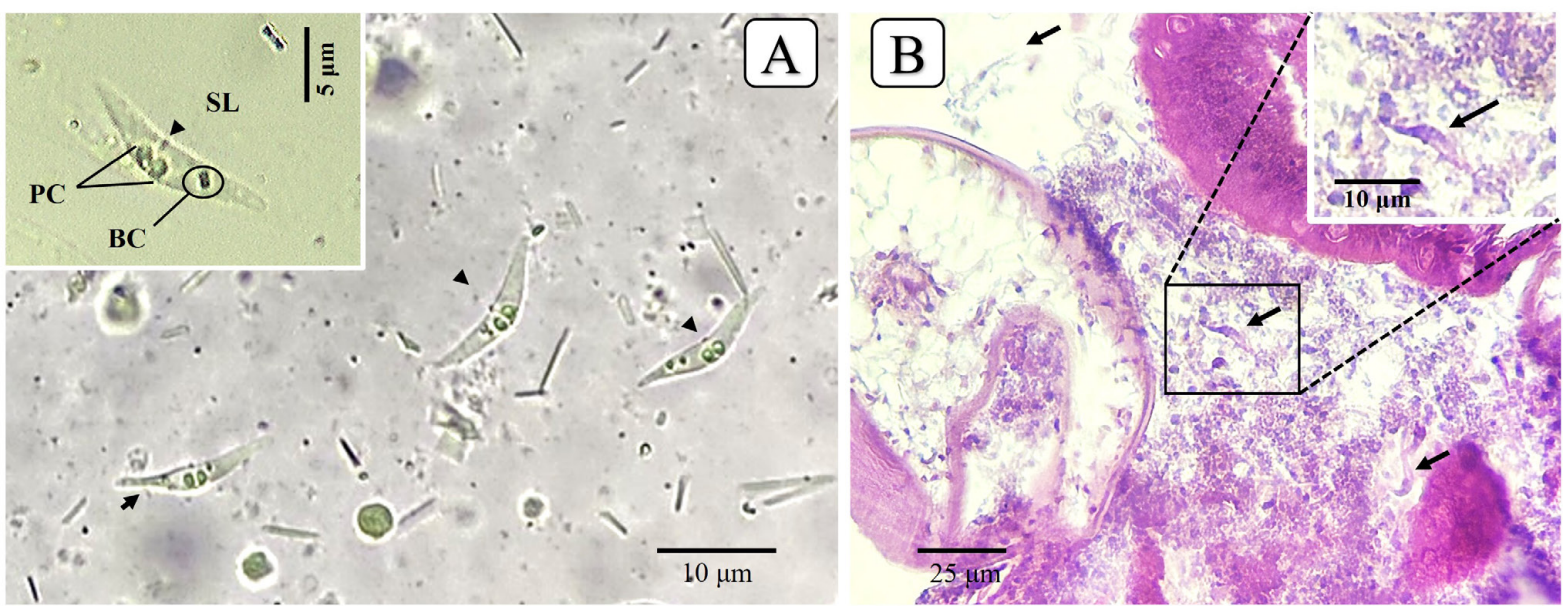
(A) Photomicrography of *Ceratomyxa tavariensis* n. sp. (black arrow), the fresh spores in the gallbladder of *Pterophyllum scalare*. PC: polar capsule; BC: binucleate sporoplasm; SL: suture line; (B) Histological section of the fish gallbladder showing the spores (black arrow) stained with Ziehl-Neelsen.

Species *Ceratomyxa tavariensis* n. sp. (Figure 2)

Host type: *Pterophyllum scalare* (Schultze, 1823)

Site of infection: Gallbladder

Type locality: Tartarugalzinho River (01°30’32.2” N 050°55’09.9” W), Tartarugalzinho municipality, Amapá State. Other locality: Farmed fish originally caught in the Pedreira River (0°28'22.8”N 50°54'21.6”W), Mangabeira community, rural area of Macapá municipality, Amapá, Brazil.

Prevalence: Six of 45 (13.3%) in Tartarugalzinho River, Tartarugalzinho municipality; 03 of 10 (30%) in Macapá municipality, Brazil; Nine of 55 (16.4%) in the state of Amapá.

Histopathology: No histological alterations were observed in the analyzed fish.

Deposited material: A representative sample of myxospores of *Ceratomyxa tavariensis* n. sp. stained with Ziehl Neelsen was deposited in the collection of the Amazon Research Institute (INPA), Manaus, Amazonas State, Brazil, under accession number: INPA-CND 000100.

Molecular data: Partial sequence of SSU rDNA with 812 bp and GenBank accession number PP994830.

Etymology: The specific epithet was given in honor of Prof. Dr. Marcos Tavares Dias, a renowned researcher in Brazil, for his great contribution to ichthyoparasitology studies.

Spore description: Morphometric description revealed mature myxospores floating freely in the bile, with a slightly curved valve (Figure 2). The myxospores measured 1.6 ± 0.2 µm in length and 11.5 ± 1.1 µm in thickness. Two subspherical polar capsules of equal size were 0.7 ± 0.1 µm in length and 0.6 ± 0.1 µm in width, located at the same level at the anterior pole of the myxospores, with 3 to 4 turns of the polar filament. Posterior angle slightly convex, with 124.2° ± 15.4 (Table 1). The suture line is noticeable between the two polar capsules and presents the binucleate sporoplasm that is located close to the polar capsule. Plasmodia, polysporic, vermiform in shape, presenting slow, undulating motility.

**Table 1:** Morphometric comparison between *Ceratomyxa tavariensis* n. sp. and *Ceratomyxa* spp. species infecting the gallbladder of fish from Brazil.

Species	Host	GenBank acession n°	Spore dimensions (µm)				PA (°)	N° of coins	References
			SP	ST	PCL	PCW			
***Ceratomyxa tavariensis* n. sp.**	*Pterophyllum scalare*	PP994830	1.6 ± 0.2	11.5 ± 1.1	0.7 ± 0.1	0.6 ± 0.1	124.2° ± 15.4	03/abr	Present study
*C. amazonensis*	*Symphysodon discus*	KX236169	7.0 ± 0.3	15.8 ± 0.4	3.2 ± 0.3	2.6 ± 0.2	105.0-115.0°	03/abr	Mathews et al. (2016)
*C. amazonensis*	*Symphysodon discus*	MN064752	4.7 ± 0.1	24.2 ± 0.4	2.2 ± 0.1	2.3 ± 0.1	154.0°	03/abr	Sousa et al. (2021)
*C. amazonensis*	*Geophagus altifrons*	OR142123	4.9 ± 1.4	23.8 ± 5.9	2.4 ± 0.8	1.9 ± 0.3	159.7° ± 10.6	03/abr	Figueredo et al. (2023)
*C. brasiliensis*	*Cichla monoculus*	KU978813	6.3 ± 0.6	41.2 ± 2.9	2.6 ± 0.3	2.5 ± 0.4	147.0°	03/abr	Zatti et al. (2017)
*C. ranunculiformis*	*Plagioscion squamosissimus*	OQ701120	4.9 ± 0.9	37.6 ± 5.2	2.0 ± 0.6	1.9 ± 0.5	165.0° ± 11.0	02/mar	Zatti et al. (2023)
*C. barbata*	*Rhaphiodon vulpinus*	MZ504286	2.9 ± 0.5	21.7 ± 3.5	1.6 ± 0.3	1.4 ± 0.2	164.0° ± 10.8	3	Franzolin et al. (2022)
*C. macapaensis*	*Mesonauta festivus*	MT939250	4.2 ± 0.5	22.8 ± 0.3	1.6 ± 0.1	1.9 ± 0.3	-	03/abr	Bittencourt et al. (2022)
*C. vermiformis*	*Colossoma macropomum*	KX278420	4.5 ± 0.2	8.4 ± 0.4	2.7 ± 0.1	2.7 ± 0.1	30.2° ± 6.6	03/abr	Adriano & Okamura (2017)
*C. fonsecai*	*Hemiodus unimaculatus*	MK796248	2.6 ± 0.1	28.9 ± 2.7	1.9 ± 0.3	1.7 ± 0.2	164.8° ± 8.6	03/abr	Silva et al. (2020)
*C.* cf. *fonsecai*	*H. orthonops*	MW053456	3.3 ± 0.2	28.0 ± 1.7	1.6 ± 0.3	1.5 ± 0.3	166.0° ± 7.4	-	Zatti et al. (2022)
*C. gracillima*	*Brachyplatystoma rousseauxii*	KY934184	4.4 ± 0.4	7.0 ± 0.5	1.9 ± 0.3	1.9 ± 0.3	37.0° ± 2.9	02/mar	Zatti et al. (2018)
*C. mandii*	*Pimelodina flavipinnis*	MZ504285	4.6 ± 0.5	31.2 ± 2.3	1.8 ± 0.3	1.9 ± 0.3	162.0° ± 10.4	03/abr	Araújo et al. (2022)
*C. microlepis*	*Hemiodus microlepis*	-	5.2 ± 0.4	35.5 ± 0.9	2.2 ± 0.3	2.2 ± 0.3	58.0-60.0º	05/jun	Azevedo et al. (2013)
*C. matosi*	*Boulengerella cuvieri*	PP791852	5.2 ± 0.3	24.5 ± 0.4	1.8 ± 0.2	1.8 ± 0.2	-	04/mai	Martel et al. (2024)
*C. edilsonis*	*Pimelodella cristata*	OR142186	1.64±0.6	17.13±2.6	1.36±0.17	0.9±0.05	152.6 ± 13.6	04/mai	Carvalho et al. (2024)

SP: spore length; ST: spore thickness; PCL: polar capsule length; PCW: polar capsule width; PA: posterior angle; trace: data not evaluated.

### Molecular identification and phylogenetic analysis

**Figure 3:**
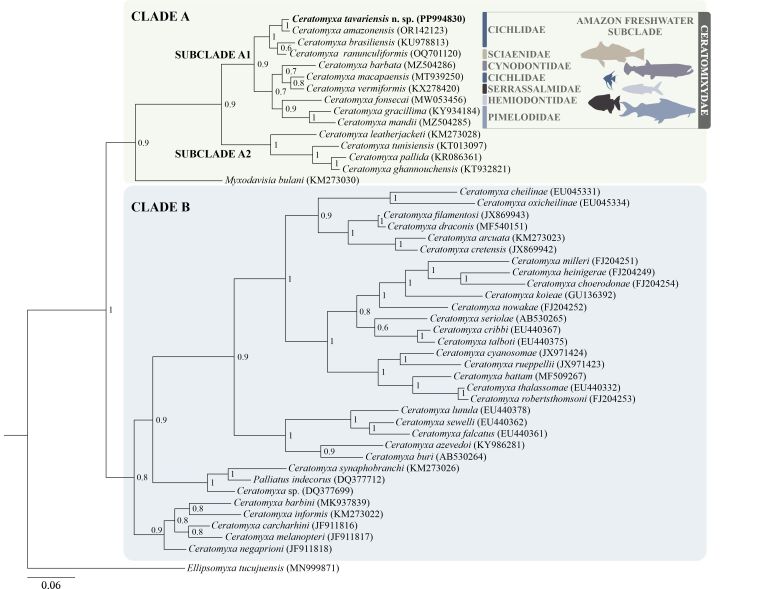
Maximum likelihood phylogenetic tree based on SSU-rDNA sequences of *Ceratomyxa tavariensis* n. sp. and other myxozoans. *Ellipsomyxa tucujuensis* was used as an outgroup. Nodal supports are indicated for Bayesian inference with posterior probabilities and are shown at each node. GenBank accession numbers are presented after each species name.

The partial 812-bp sequence of the SSU-rDNA gene was obtained from the sequencing of a *Ceratomyxa* species. The SSU rDNA molecular markers formed clades A and B with strong nodal support (BI = 1) (Figure 3). Clade A was subdivided into the following subclades (strong support, BI = 0.9): A1, which included *C. tavariensis* n. sp. and nine freshwater ceratomyxids from the Brazilian Amazon, which parasitized fish from six distinct families (Cichlidae, Sciaenidae, Cynodontidae, Serrasalmidae, Hemiodontidae and Pimelodidae); and subclade A2 grouped marine ceratomyxid species. Clade B included *Ceratomyxa* spp. from marine environments. *Ceratomyxa tavariensis* n. sp. found in the gallbladder of the cichlid *P. scalare* grouped with the sister species that included species that infect Cichlidae and Sciaenidae hosts. However, *Ceratomyxa macapaensis* Bittencourt et al., 2022, a parasite that also occurs in the state of Amapá, did not appear in the phylogenetic branch of *Ceratomyxa* of Cichlidae hosts.

The BLASTn search revealed no identical correspondence between these sequences and any other SSU-rDNA sequence available in GenBank, and the minimum genetic distance (p) was 1.02% between *C. tavariensis* n. sp. and *C. amazonensis* Mathews, Naldoni, Maia & Adriano, 2016 (OR142123) (Table 2). The other sequences recovered distances greater than 2.44% and the largest genetic distance was 9.06% for the species *Ceratomyxa mandii* Araújo, Adriano, Franzolin, Zatti & Naldoni, 2022.

**Table 2:** Comparative genetic distance between *Ceratomyxa tavariensis* n. sp. and freshwater *Ceratomyxa* species from the Brazilian Amazon.

	**Species**	**1**	**2**	**3**	**4**	**5**	**6**	**7**	**8**	**9**	**10**
1	*Ceratomyxa tavariensis* n. sp.										
2	*Ceratomyxa amazonensis*	1.02									
3	*Ceratomyxa brasiliensis*	3.32	3.20								
4	*Ceratomyxa ranunculiformis*	2.44	2.44	2.57							
5	*Ceratomyxa barbata*	5.04	5.30	6.31	5.70						
6	*Ceratomyxa macapaensis*	4.88	4.89	5.68	5.67	4.53					
7	*Ceratomyxa vermiformis*	5.64	5.25	5.78	4.90	4.02	2.06				
8	*Ceratomyxa fonsecai*	7.28	7.16	7.78	6.88	7.50	6.54	6.01			
9	*Ceratomyxa gracillima*	8.21	7.70	7.81	8.34	8.57	6.42	6.28	7.72		
10	*Ceratomyxa mandii*	9.06	8.93	9.57	8.17	8.96	7.06	6.41	8.95	5.62	

## Discussion

Based on the morphological characteristics, the observed spores were consistent with those defined for the genus *Ceratomyxa*, according to the generic description proposed by Lom & Dyková (2006) and Heiniger & Adlard (2013). This study provides the morphological description combined with SSU rDNA sequences for the new Myxozoan species, *Ceratomyxa tavariensis* n. sp., parasitic of the ornamental fish *P. scalare*.

*Pterophyllum scalare* is known to be a host to some parasites, including crustacean arthropod species, monogeneans, nematodes and protozoa (Fujimoto et al., 2006; Neves & Tavares-Dias, 2019; Rahmati-Holasoo et al., 2022; Santos et al., 2024). However, the presence of myxozoans infecting this species had not been previously reported.

When comparing the morphology of the spores of *C. tavariensis* n. sp. with the species occurring in Brazil, a greater affinity was demonstrated with *C. amazonensis* (Figueredo et al., 2023), a parasite of *Geophagus altifrons* Heckel, 1840 (Cichlidae) collected in the Tapajós River, near the municipality of Santarém-PA, Brazil. However, the new species exhibited morphometric differences from *C. amazonensis* by presenting short and narrow spore dimensions (1.6 ± 0.2 x 11.5 ± 1.1 µm for *C. tavariensis* n. sp. and 4.9 ± 0.1 x 23.8 ± 5.9 µm for *C. amazonensis*). Differences were also observed in the dimensions of the polar capsules, with *C. tavariensis* n. sp. smaller in length and width (0.7 ± 0.1 × 0.6 ± 0.1 µm in the present study and 2.4 ± 0.8 × 1.9 ± 0.3 µm in *C. amazonensis*), in addition to diverging in region of occurrence (state of Amapá for *C. tavariensis* n. sp. and states of Amazonas and Pará for *C. amazonensis*) and host fish genus (*Pterophyllum* for *C. tavariensis* n. sp. and *Symphysodon* and *Geophagus* for *C. amazonensis*). In the present study, the morphometric data of *Ceratomyxa* parasitizing hosts originating from the municipality of Tartarugalzinho converge with those observed in the municipality of Macapá, state of Amapá.

Regarding the specificity of parasite-host interactions, *Ceratomyxa* species showed high specificity for host species that inhabit restricted areas of endemism in the Amazon Basin. Host ecological characteristics can influence both parasite endemism and radiation of myxosporidian parasites in the Amazon Basin (Mathews et al., 2020; Zatti et al., 2018). For example, Zatti et al. (2018) described *Ceratomyxa gracillima* Zatti, Atkinson, Maia, Bartholomew & Adriano, 2018 in migratory freshwater catfish sampled from geographically distant areas in the Amazon Basin and concluded that host migration can lead to radiation of Amazonian ceratomyxids. Furthermore, Bittencourt et al. (2022) observed *Ceratomyxa macapaensis* Bittencourt et al., 2022 in the cichlid *Mesonauta festivus* widely distributed in the state of Amapá. However in the present study this specific *Ceratomyxa* species was not detected in *P. scalare*, a non-migratory fish with asynchronous gonadal development, belonginh tothe Cichlid family and collected in the same geographic area.

In Brazil, *Ceratomyxa* species specifically infect the gallbladder. Although ceratomyxids have been shown to have tropism for host tissue, the species *Ceratomyxa qingdaoensis* Zhao, Al-Farraj, Al-Rasheid & Song, 2015 was found in the urinary bladder of *Argyrosomus argentatus* collected in coastal waters of China (Gunter et al., 2010; Zhao et al., 2015).

The BLASTn search showed that *C. tavariensis* n. sp. diverged from the sequences available in GenBank. Investigations that deal with genetic sequencing generally accept differences of around 1% to establish new species of myxozoans, and these identifications must be relatedto other taxonomic characters, such as myxospore morphometry, host species and tissue specificity (Atkinson et al., 2015; Bartošová-Sojková et al., 2018; Rocha et al., 2023).

The SSU rDNA molecular markers of this genus were grouped into two well-defined clades, with primary division of phylogenies according to host habitat, with clade A grouping mainly freshwater species and clade B exclusively marine species (Fiala, 2006; Kent et al., 2001). In this study, the arrangement of *Ceratomyxa* species showed the same behavior as other phylogenetic studies, with the new species being inserted into the monophyletic subclade composed of freshwater species occurring in the Amazon (Fiala et al., 2015; Zatti et al., 2023). However, the presence of marine ceratomyxids (*C. tunisiensis* Thabet, Mansour, Al Omar & Tlig‐Zouari, 2015, *C. leatherjacketi* Fiala, Hlavničková, Kodádková, Freeman, Bartošová-Sojková & Atkinson, 2015, *C. pallida* Thélohan, 1895 and *C. ghannouchensis* Thabet, Abdel-Baki, Harrath & Mansour, 2019) grouped in subclade A2 basally within the freshwater lineage is possibly explained by the type of definitive host being the main factor relating the lineages of these parasites (Fiala et al., 2015; Holzer et al., 2007).

It was noticeable that *C. tavariensis* n. sp. aligned with the phylogenetic subclade A1 of teleost fish parasites that inhabit freshwater. In Brazil, most freshwater *Ceratomyxa* species were found in the Amazon River basin and few studies have been developed in the Paraná River basin and the Tocantins River basin (Franzolin et al., 2022; Silva et al., 2020; Zatti et al., 2022). Future research on myxozoans of host fishes inhabiting poorly investigated basins may contribute to expanding knowledge of the ichthyoparasitic diversity of the Myxozoa Class in Brazil. The occurrence of freshwater *Ceratomyxa* infecting fishes in other continents was recently reported by Li et al. (2023), when studying the yellow catfish (*Trachysurus fulvidraco* Richardson, 1846), a fish of commercial importance in China, and demonstrated genetic similarity with strictly marine ceratomyxid species.

Currently, eleven species of ceratomyxids have been described infecting wild fish from Brazil (Araújo et al., 2022; Bittencourt et al., 2022; Eiras et al., 2018; Franzolin et al., 2022; Silva et al., 2020; Zatti et al., 2023). *C. tavariensis* n. sp. is the thirteenth *Ceratomyxa* species described from fish in the country. Furthermore, this was the first morphomolecular investigation of the diversity of myxosporidian worms infecting *P. scalare* distributed in the state of Amapá.

## Conclusion

Overall, this integrative taxonomic investigation provides new information on the characteristics of the ichthyoparasitic fauna of *P. scalare*. The present work represents the first description of *C. tavariensis* n. sp. in angelfish in Brazil, demonstrated through morphological, morphometric and molecular characters. *Ceratomyxa tavariensis* n. sp. was detected in the gallbladder of *P. scalare*, with low prevalence (16.4%) in the state of Amapá.

To complement this investigation, it is recommended to study myxozoan ichthyoparasites for important export species for the ornamental industry in Brazil that can be vectors of pathogens, in addition to pointing out the potential zoonotic risk of myxozoans.
